# *De Novo* Transcriptome Analysis of Two Seahorse Species (*Hippocampus erectus* and *H*. *mohnikei*) and the Development of Molecular Markers for Population Genetics

**DOI:** 10.1371/journal.pone.0154096

**Published:** 2016-04-29

**Authors:** Qiang Lin, Wei Luo, Shiming Wan, Zexia Gao

**Affiliations:** 1 Key Laboratory of Tropical Marine Bio-resources and Ecology, South China Sea Institute of Oceanology, Chinese Academy of Sciences, Guangzhou, Guangdong, 510275, China; 2 Key Laboratory of Agricultural Animal Genetics, Breeding and Reproduction of Ministry of Education/Key Lab. of Freshwater Animal Breeding, Ministry of Agriculture, College of Fisheries, Huazhong Agricultural University, Freshwater Aquaculture Collaborative Innovation Center of Hubei Province, Wuhan, 430070, China; Shanghai Ocean University, CHINA

## Abstract

Seahorse conservation has been performed utilizing various strategies for many decades, and the deeper understanding of genomic information is necessary to more efficiently protect the germplasm resources of seahorse species. However, little genetic information about seahorses currently exists in the public databases. In this study, high-throughput RNA sequencing for two seahorse species, *Hippocampus erectus* and *H*. *mohnikei*, was carried out, and *de novo* assembly generated 37,506 unigenes for *H*. *erectus* and 36,113 unigenes for *H*. *mohnikei*. Among them, 17,338 (46.23%) unigenes for *H*. *erectus* and 17,900 (49.57%) for *H*. *mohnikei* were successfully annotated based on the information available from the public databases. Through comparing the unigenes of two seahorse species, 7,802 candidate orthologous genes were identified and 5,268 genes among them could be annotated. In addition, gene ontology analysis of two species was similarly performed on biological processes, cellular components, and molecular functions. Twenty-four and twenty-one unigenes in *H*. *erectus* and *H*. *mohnike*i were annotated in the biosynthesis of unsaturated fatty acids pathways, and both seahorses lacked the Δ12 and Δ15 desaturases. Total of 8,992 and 9,116 SSR loci were obtained from *H*. *erectus* and *H*. *mohnikei* unigenes, respectively. Dozens of SSR were developed and then applied to assess the population genetic diversity, as well as cross-amplified in a related species, *H*. *trimaculatus*. The *H*_*O*_ and *H*_*E*_ values of the tested populations for *H*. *erectus*, *H*. *mohnikei*, and *H*. *trimaculatus* were medium. These resources would facilitate the conservation of the species through a better understanding of the genomics and comparative genome analysis within the *Hippocampus* genus.

## Introduction

Seahorses belong to the family Syngnathidae, genus *hippocampus*, which is an iconic and ecologically important species globally, and so far they are relevant in many issues in marine conservation [1, 2]. Seahorses are frequently captured as trawl by catch, and their habitats are vulnerable to destruction, which leads to great pressure on wild stocks of seahorses [1, 3]. The heavy trade of seahorses for the Chinese traditional medicine, and ornamental and curio markets has caused a dramatic decline in many tropical source regions throughout the world [4, 5]. Therefore, in Endangered Species of Wild Fauna and Flora, seahorses have been listed in Appendix II of the Convention on International Trade [6].

Fortunately, seahorse aquaculture sprung up over the past ten years has been deemed as an effective strategy to partially decrease fishing pressure on wild seahorse population [2, 7–9]. However, the low germplasm resources of seahorses are still restricting the development of seahorse culture [10]. Developing predominant varieties and improving aquaculture techniques for seahorses are the important strategies to relieve the stress imposed by overfishing. So far, the relevant researches mainly focus on the biology [11], conservation [1], and aquaculture of seahorse species [9, 12, 13]. One of the key obstacles for selective breeding of seahorses is the lack of knowledge of their transcriptomic or genomic information, and availability of molecular markers.

In the recent years, the high-throughput sequencing technology has become a quite effective approach for a large amount of genes discovery on a genome-wide scale for non-model organisms [14]. RNA-seq and DNA-seq have opened the way to study functional and genetic information for many organisms [15]. As to a species of interest with no or little available genomic resources, transcriptome sequencing could offer a cost-effective method for high-throughput genes discovery. Compared to the full genome sequencing projects, transcriptome sequencing projects are more computationally tractable, but still can generate sufficient resources to meet the requirements of many non-model species’ researching projects [16].

Microsatellites (SSRs), which have many useful properties, such as good reproducibility, high polymorphism and codominant inheritance, have been widely used in population genetics, germplasm resource analysis [15–18] and molecular assisted selection (MAS) [19, 20]. Currently, only a few genetic markers are available for seahorses, including mitochondrial DNA (mtDNA) [21, 22] and microsatellites [23–25] developed by traditional methods. These data, however, have partially limited applicability for the breeding and germplasm improvement of seahorses.

The conservation and management of seahorse populations are of great international concern [26]. Among the seahorse species, the lined seahorse (*Hippocampus erectus*) and Japanese seahorse (*H*. *mohnikei*) have been suggested as the candidate species for aquaculture [2, 5, 8]. These two seahorse species are differed significantly from their morphology and distributions. *H*. *erectus* is larger in body size than that of *H*. *mohnikei* and it exists widely in the western Atlantic Ocean, and especially the area around the Gulf of Mexico, and *H*. *mohnikei* is distributed around the area of the North-western Pacific Ocean [1, 27]. Due to the insufficiency of available genomic or transcriptomic resources in the genus *Hippocampus*, Illumina Solexa sequencing technology was utilized to characterize the transcriptomes of *H*. *erectus* and *H*. *mohnikei*, and then the SSR markers were developed according to the transcriptome data to study the population genetics in seahorses.

## Materials and Methods

### Sample collection and preparation

*H*. *erectus* were sampled from a cultured population in Florida, United States (n = 40), and all the seahorses were cultured in indoor re-circulating holding tanks (90×80×60 cm) at Vero Beach marine laboratory, and the samples were approved only for the experimental usage by Florida Institute of Technology. *H*. *mohnikei* were collected from Yangmadao, China (n = 27), and *H*. *trimaculatus* were sampled along the China’s coast (n = 72). All the locations for seahorses sampling were absolutely approved that there were no any endangered or protected species by the local Marine and Fishery Bureaus (A201201I03) and Chinese Academy of Sciences (KZCX2-EW-QN206). Besides a few specimens (n = 17) were obtained with the help of the local fishers and buyers, most samples were captured by researchers using trawl boats. Cyt *b* and COI sequences from the tissue of dorsal fin (0.3×0.4 cm) of the seahorses were used to complete the species identifications before the further study [28], and all specimens were kept in 95% alcohol to extract genomic DNA for the evaluation of genetic diversity of seahorses (Table 1). The seahorses utilized in this study have been fully approved for the use of research work by the South China Sea Institute of Oceanology, Chinese Academy of Sciences (SCSIO-CAS). All samples used have received animal ethics approval for experimentation by the CAS (2011250).

**Table 1:** Sampling location and sample size (n) of *H*. *erectus*, *H*. *mohnikei* and *H*. *trimaculatus*. *H*. *erectus* were cultured and approved only for the experimental usage by Florida Institute of Technology (FIT).

Species	Location	Latitude	Longitude	n	Provider
*H*. *erectus*	Vero Beach, FL, USA	27.39°N	80.23°W	40	Researcher in FIT
*H*. *mohnikei*	Yangmadao	37.50°N	121.65°E	27	Researcher
*H*. *trimaculatus*	Fuzhou	26.08°N	119.14°E	23	Researcher
_	Quanzhou	24.53°N	118.36°E	3	Buyer
_	Shantou	23.02°N	116.28°E	16	Researcher
_	Yangjiang	21.65°N	112.20°E	1	Researcher
_	Haikou	20.03°N	110.13°E	4	Fisher
_	Qinghai	19.25°N	110.46°E	15	Researcher
_	Sanya	18.15°N	109.27°E	10	Fisher

The obtained seahorse samples for sequencing were sponsored by Zhanjiang Seahorse Center of the SCSIO-CAS, and the seahorses were kept in recirculating holding tanks (70×50×40 cm), and the seawater was from South China sea and treated with double sand filtration with the temperature 26±2°C, pH 8.1±0.2, and salinity of 31±0.5‰ for two weeks prior to sampling. All the seahorses were fed twice a day (0900 and 1400 h) with frozen *Mysis* sp. and live *Artemia* and uneaten food were siphoned out daily. One year old *H*. *erectus* and *H*. *mohnikei* were used for cDNA library construction through the euthanasia. Samples of the tissues, including brain, pituitary and ovary (development at stage V) were collected from three seahorses for each species, and all the seahorses were sacrificed after being anaesthetized for 30 min by MS-222 (Vivify Nature, China), which is approved for fish by FDA (Food and Drug Administration, USA). The sampling method was approved by the equivalent animal ethics committee of the CAS, and all the sampling procedures were specifically approved as part of obtaining the field permit. All samples were frozen immediately in liquid nitrogen, and stored at -80°C until use.

### RNA isolation

Three seahorses in each species were used for RNA isolation, and fresh tissues including brain, pituitary and ovary were dissected and isolated RNA immediately. Total RNA was isolated using TRIzol reagent according to the manufacturer’s instructions (Invitrogen). The quantity and quality of total RNA of each tissue sample was measured using a Nanodrop 2000 (Thermo Scientific) and Agilent 2100 Bioanalyzer (Agilent Technologies). For each species, the 0.50 μg RNA from each tissue of brain, pituitary and ovary was combined and mixed into a single pool for the final usage. The RNA pools were DNase-treated using Turbo DNA-free (Ambion) and then purified with RNeasy Mini Kit (QIAGEN).

### cDNA library preparation and sequencing

The construction of the libraries and the RNASeq were performed by Shanghai OE Biotech Company (Shanghai, China). The poly (A) mRNA purification, mRNA fragmentation and the cDNA library preparation for transcriptome sequencing were conducted using TruSeq RNA Sample Preparation kit v2 (Illumina Inc. San Diego, CA, USA; Catalog IDs 15025062). Then, the paired-end cDNA library with an insert size of 200 bp was prepared in accordance with Illumina’s protocols. The cDNA libraries were sequenced on the Illumina HiSeq2000 genomic sequencer platform at Shanghai OE Biotech Company, China. The sequencing of *H*. *erectus* and *H*. *mohnikei* were performed respectively.

### Transcriptome data processing and assembly

For assembly, the clean reads were obtained through removing raw reads with adaptor and unknown nucleotides above 5% or those that were of low quality (containing more than 50% based with Q-value < = 20) were removed to obtain clean reads using a custom Perl script by Shanghai OE Biotech Company, China. Then the clean sequence data obtained for *H*. *erectus* and *H*. *mohnikei* were used to do *de novo* assembly, respectively. The Trinity software [14] (http://trinityrnaseq.sourceforge.net/, Version r2013_08_14) were used for *de novo* transcriptome assembly with default parameters using Kmer = 25, which had been used in many publications related to transcriptome data analysis [15, 16]. The contigs and unigenes with length of less than 200 bp were discarded due to a low annotation rate [16, 29]. The clean reads of the *H*. *erectus* and *H*. *mohnikei* were submitted to the NCBI Sequence Read Archive under the accession number of SRX535337, with SRR1275087 for *H*. *erectus* and SRR1280030 for *H*. *mohnikei*. The assembled unique sequences were deposited in the NCBI TSA DataSets under accession number of SUB531774.

### Functional annotation, CDS prediction and orthologous genes identification

To annotate the transcriptomes of *H*. *erectus* and *H*. *mohnikei*, functional annotations were performed by sequence comparison with public databases included the NCBI non-reduntant protein database (Nr, by December 2014) (http://www.ncbi.nlm.nih.gov), Swiss-Prot database (http://www.expasy.ch/sprot), Clusters of orthologous groups for eukaryotic complete genomes database (KOG) (ftp://ftp.ncbi.nih.gov/pub/COG/KOG/kyva) and Kyoto encyclopedia of genes and genomes (KEGG) pathway database (http://www.genome.jp/kegg/) using BLASTX alignment with an E-value of 1e^-5^, respectively. Based on the results of Nr database annotation, Blast2GO program [30] were used to perform GO annotation of unigenes and then WEGO [31] software was used to perform GO classification, describing biological processes, molecular functions and cellular components for every transcript.

The coding sequences (CDS) for unigenes were predicted by BLASTX and ESTscan. The transcript sequences were searched against the Nr, KOG, KEGG and Swiss-Prot protein databases using BLASTX (e-value < 10^−5^). The best alignment results were used to determine the sequence direction of unigenes. When a transcript could not be aligned to any database, ESTScan Program [32] was used to predict coding regions and determine sequence direction.

The putative orthologous genes between the unigenes of *H*. *erectus* and *H*. *mohnikei* were searched by BLASTN program of BLAST 2.2.28+ software with E-value of 1e^-5^. Through using the reciprocal best hit method with the BLASTN algorithm, the two unigenes from *H*. *erectus* and *H*. *mohnikei* with the best BLAST results (≥ 60%) were taken as the candidate orthologous genes.

### SSRs identification and cross-amplification

All the obtained unigenes were used to search for microsatellite makers using MISA program [33] based on the criteria of a repeat threshold of six for di-, five for tri-, tetra-, penta- and hexa-nucleotide repeats. Considered the requirements for primer design, the sequences without containing 50-bp sequence on both sides of the microsatellite repeat were discarded for further analysis.

To validate the SSR makers, 10 individuals of two species were used at 87 and 94 random SSRs loci for *H*. *mohnikei* and *H*. *trimaculatus*, respectively. Total DNA was isolated from the dorsal fin of seahorse by using the Phenol/Chloroform procedure. PCR amplifications were conducted in a final volume of 10 μL containing 50 ng templates DNA, 1× PCR buffer, 2.0 mM MgCl_2_, 2.5 mM dNTPs, 2.5 μM of each primer, and 1 U Taq polymerase. The PCR reaction cycling profile was 95°C for 5 min followed by 30 cycles of 35 s at 95°C, 40 s at primer-specific annealing temperature, 45 s at 72°C, and a final extension at 72°C for 8 min. The amplified PCR products were separated on 8% non-denaturing polyacrylamide gels at 150 V lasting 2 h and visualized via silver-staining.

To determine the cross utility of microsatellite markers developed in this study, cross-species amplification of the identified markers was assessed between *H*. *mohnikei* and *H*. *trimaculatus*. The identified markers for *H*. *mohnikei* and *H*. *trimaculatus* were also tested in another related seahorse’s species, *H*. *trimaculatus*.

### Genetic diversity analysis in seahorses

The identified polymorphic SSR markers were employed to analyze the genetic diversity of *H*. *erectus*, *H*. *mohnikei*, and *H*. *trimaculatus* populations. The software Popgene 32 was utilized to calculate the number of alleles (*N*_*A*_), observed heterozygosity (*H*_*O*_), expected heterozygosity (*H*_*E*_), and Hardy-Weinberg equilibrium (HWE). The results were adjusted for multiple simultaneous comparisons using a sequential Bonferroni correction. Polymorphism information content (PIC) values were calculated using the formula PIC = 1- Σ*P*_*i*_^2^, in which *P*_*i*_ is the frequency of the *i*th allele [32].

## Results

### Illumina sequencing and de novo assembly

The total of 50,948,904 and 49,476,082 raw sequencing reads were generated for *H*. *erectus* and *H*. *mohnikei* using Illumina paired-end sequencing technology, respectively. After quality control of raw data, which removing and trimming the low-quality reads, adapters, poly-A tails, and reads containing more than 5% unknown nucleotides, approximately 4,090,677,279 bp and 3,960,288,422 bp of high-quality data were achieved, yielding total of 42,642,934 and 41,331,060 clean reads for *H*. *erectus* and *H*. *mohnikei*, respectively. The overview of the sequencing and assembly statistics are shown in Table 2.

**Table 2:** Summary of Illumina paired-end sequencing and assembly for *H*. *erectus* and *H*. *mohnikei*.

Database	*H*. *erectus*		*H*. *mohnikei*	
	Number	Total length (bp)	Number	Total length (bp)
Total raw reads	50,948,904	5,094,890,400	49,476,082	4,947,608,200
Total clean reads	42,642,934	4,090,677,279	41,331,060	3,960,288,422
Number of contigs	337,628	673,692,247	283,894	463,004,720
Average length of contigs (bp)	1,995		1,631	
Max length of contigs (bp)	23,393		24,257	
Min length of contigs (bp)	201		201	
Contig size N50 (bp)	3,572		3,004	
Number of unigenes	37,506	49,413,208	36,113	48,204,820
Average length of unigenes (bp)	1,317		1,335	
Max length of unigenes (bp)	23,393		27,745	
Min length of unigenes (bp)	201		201	
Transcript size N50 (bp)	2,215		2,205	
Number of unigenes > = 1000 bp	14,855		14,891	

**Fig 1:**
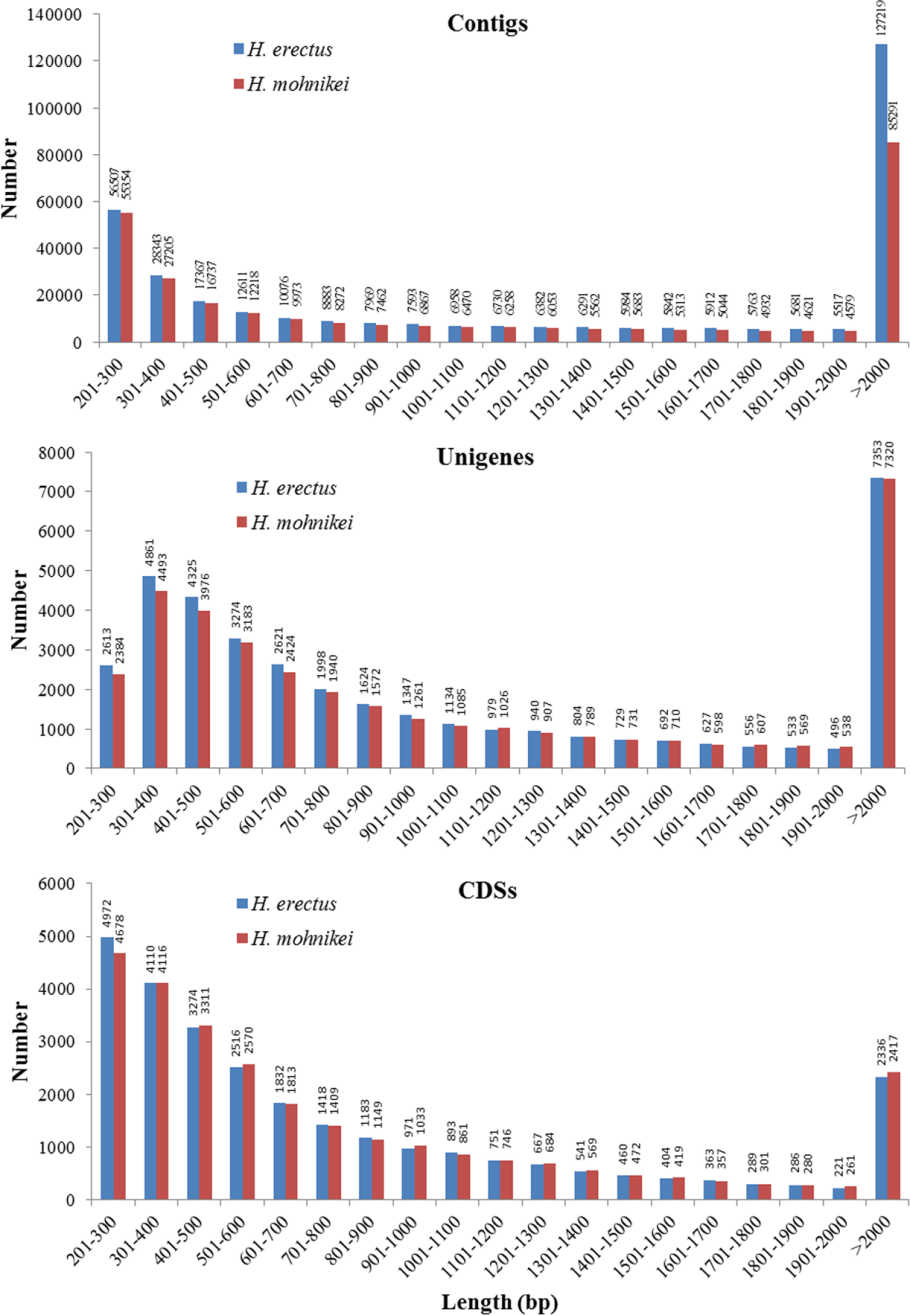
Length distribution of all contigs, unigenes and CDSs for *H*. *erectus* and *H*. *mohnikei* (Unit: bp).

The clean reads obtained for *H*. *erectus* and *H*. *mohnikei* were used to assemble the transcriptome for each species using the Trinity *de novo* assembler, respectively. The total of 337,628 contigs consisting of 673,692,247 bp for *H*. *erectus* and 283,894 contigs consisting of 463,004,720 bp for *H*. *mohnikei* were assembled firstly. In *H*. *erectus*, the size of the contigs ranged from 201 to 23,393 bp, with a mean length of 1,995 bp and N50 length of 3,572 bp (Fig 1). As to *H*. *mohnikei*, the maximum size of the contigs was 24,257 bp, with a mean length of 1,631 bp and N50 length of 3,004 bp (Fig 1). Among these contigs, 188,279 (55.77%) for *H*. *erectus* and 139,806 (49.25%) for *H*. *mohnikei* were longer than 1000 bp. These contigs were further assembled into unigenes using paired-end joining and gap-filling methods.

The total of 37,506 unigenes with a mean length of 1,317 bp for *H*. *erectus* and 36,113 unigenes with a mean length of 1335 bp for *H*. *mohnikei* were obtained in the study. There were 14,843 unigenes (39.58%) for *H*. *erectus* and 14,880 unigenes (41.20%) for *H*. *mohnikei* longer than 1,000 bp, as well as 7,353 unigenes (19.60%) for *H*. *erectus* and 7,320 (20.27%) for *H*. *mohnikei* longer than 2,000 bp. The size distribution of these unigenes was shown in Fig 1. In this study, the coding sequences (CDS) from *H*. *erectus* and *H*. *mohnikei* unigenes sequences were also detected and a total of 27,487 and 27,446 CDSs were obtained, respectively, among them, 7,211 (26.23%) and 7,367 (26.84%) CDSs were longer than 1,000 bp for *H*. *erectus* and *H*. *mohnikei*, respectively (Fig 1). These unigenes have been deposited in the NCBI Sequence Read Archive (SRA) database (accession number: SRR2922398, SRR2922418).

### Functional annotation

The total of 17,338 unigenes (46.23%) for *H*. *erectus* and 17,900 unigenes (49.57%) for *H*. *mohnikei* were annotated based on the information available from public databases including Nr, Swiss-Prot protein, KOG and KEGG using BLASTX with an E-value cut-off of < 10^−5^ (Table 3; S1 File). Among them, 5,500 unigenes for *H*. *erectus* and 5,709 unigenes for *H*. *mohnikei* showed significant matches to all four databases. Unigenes of *H*. *erectus* that were annotated in public databases are as follows: 17,268 (46.04%), 15,674 (41.79%), 13,169 (35.11%) and 6,403 (17.07%) unigenes in Nr, Swiss-Prot, KOG and KEGG, respectively. For *H*. *mohnikei*, there were 17,810 (49.32%), 16,428 (45.49%), 13,607 (37.68%) and 6,601 (18.28%) unigenes significantly matched to Nr, Swiss-Prot, KOG and KEGG among 36,113 assembled unigenes, respectively, which were similar to *H*. *erectus*. Furthermore, about 53.77% of unigenes (20,168) for *H*. *erectus* and 50.43% of unigenes (18,213) for *H*. *mohnikei* did not show any matches to known genes.

**Table 3:** Summary of annotation percentage of *H*. *erectus* and *H*. *mohnikei* unigenes as compared to public database.

Database	*H*. *erectus*		*H*. *mohnikei*	
	Number of unigenes	Annotation percentage (%)	Number of unigenes	Annotation percentage (%)
Nr	17,268	46.04	17,810	49.32
SwissProt	15,674	41.79	16,428	45.49
KEGG	6,403	17.07	6,601	18.28
KOG	13,169	35.11	13,607	37.68
All annotated unigenes	17,338	46.23	17,900	49.57
Total unigenes	37,506		36,113	

**Fig 2:**
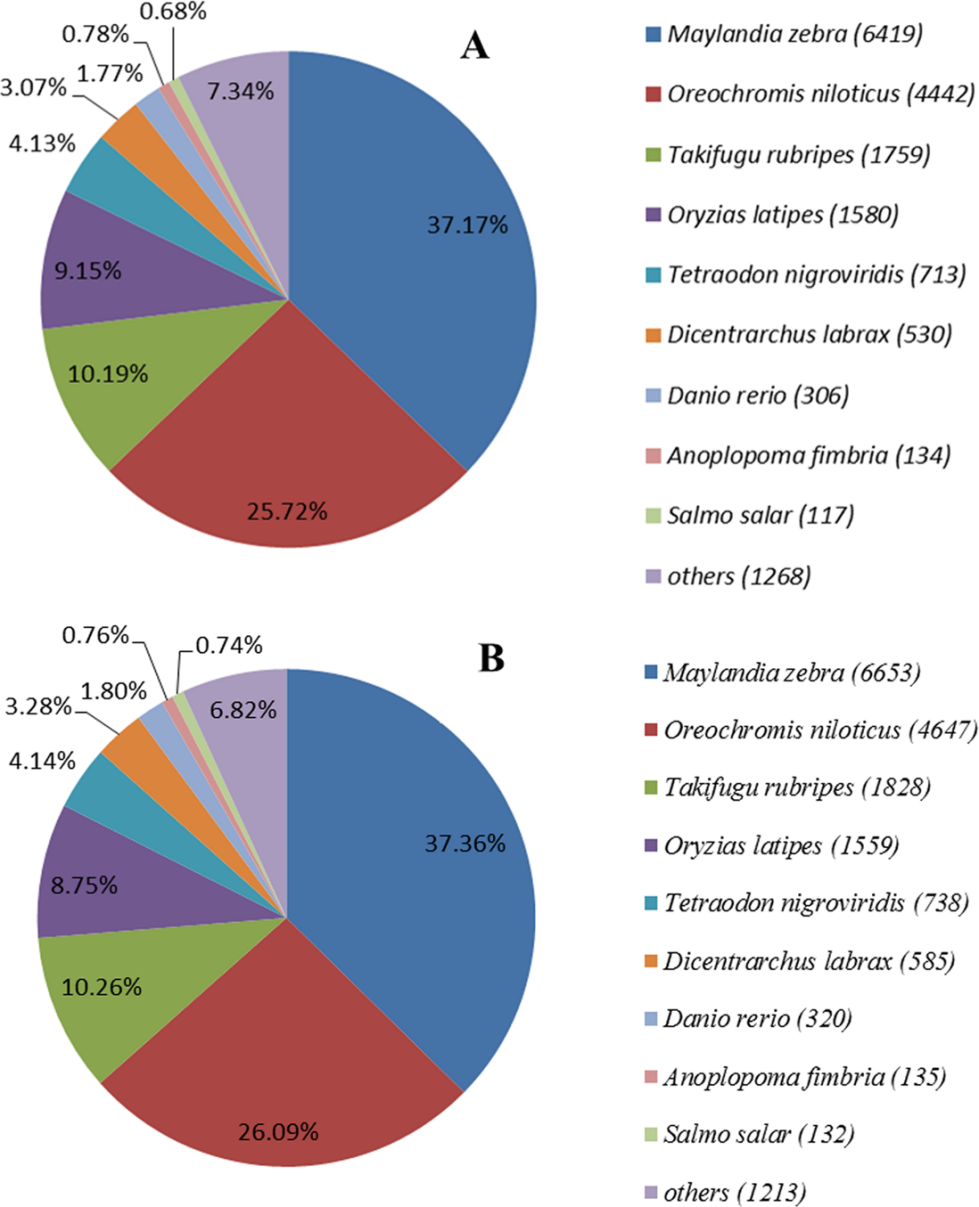
Top 9 best hits fish species distribution for homologous unigenes from *H*. *erectus* (A) and *H*. *mohnikei* (B) BLASTX against the Nr database.

Our results showed that approximately 80% of unigenes over 1,000 bp in length in the both species had BLAST matches against the Nr database; however, only about 25% of unigenes with lengths shorter than 1,000 bp in the both species generated BLAST matches (S1 File). The same tendency was also observed in BLAST results against the SwissProt database. The e-value distribution of the top hits in the Nr database revealed that 78.28% of the mapped unigenes for *H*. *erectus* and 78.57% of the mapped unigenes for *H*. *mohnikei* showed significant homology (e value < 10^−50^). The species distribution of the best matches for each sequence was as shown in Fig 2. For both species, zebra mbuna (*Maylandia zebra*) provided matches for 37.17% and 37.36% of sequences for *H*. *erectus* and *H*. *mohnikei*, respectively, followed by Nile tilapia (*Oreochromis niloticus*) (25.72% for *H*. *erectus* and 26.09% for *H*. *mohnikei*), pufferfish (*Takifugu rubripes*) (10.19% for *H*. *erectus* and 10.26% for *H*. *mohnikei*) and medaka (*Oryzias latipes*) (9.15% for *H*. *erectus* and 8.75% for *H*. *mohnikei*).

After the BLAST between the unigenes of the two seahorse species, a total of 7,802 pairs of putative orthologous genes were obtained (S2 File), using the reciprocal best hit method with the BLASTN algorithm. Among these unigenes, a total of 5,268 genes could be annotated based on the information available from public databases including Nr, Swiss-Prot, KOG and KEGG.

### Gene ontology and KOG classification

**Fig 3:**
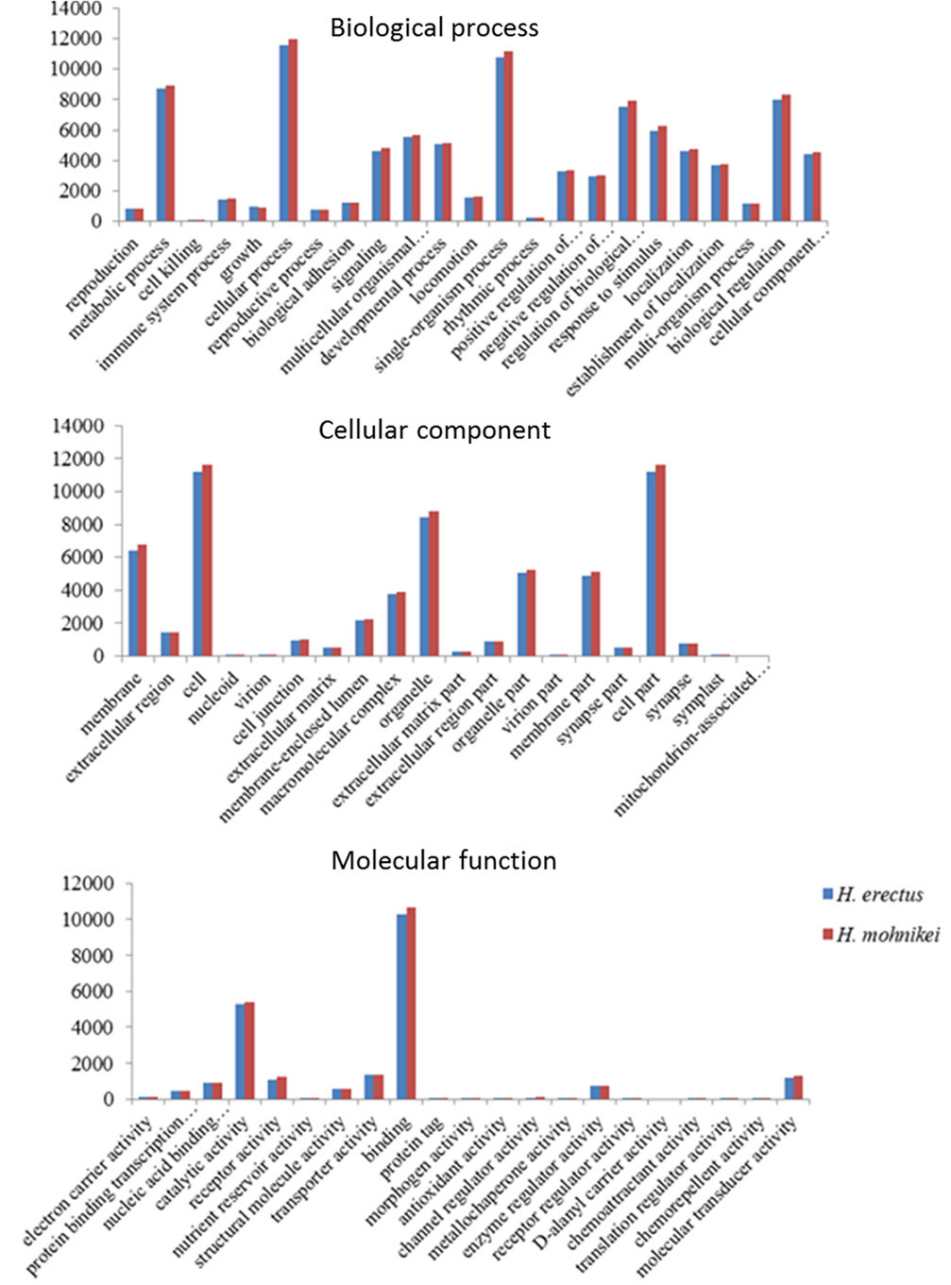
The GO classifications of assembled unigenes for *H*. *erectus* and *H*. *mohnikei*.

Based on the Nr annotation, Gene Ontology (GO) classification was used to classify the functions of all unigenes. The total of 14,645 and 15,197 unigenes were assigned to one or more GO terms for *H*. *erectus* and *H*. *mohnikei*, respectively (S3 File). These unigenes were found to be involved in biological process, cellular components and molecular functions in *H*. *erectus* and *H*. *mohnikei*, respectively (Fig 3). In both species, unigenes related to cellular process (11,574 unigenes for *H*. *erectus* and 11,986 unigenes for *H*. *mohnikei*), cell (11,194 unigenes for *H*. *erectus* and 11,655 unigenes for *H*. *mohnikei*), and binding (10,262 unigenes for *H*. *erectus* and 10,645 unigenes for *H*. *mohnikei*) were all the most abundant genes in the biological processes, cellular components, and molecular functions, respectively.

**Fig 4:**
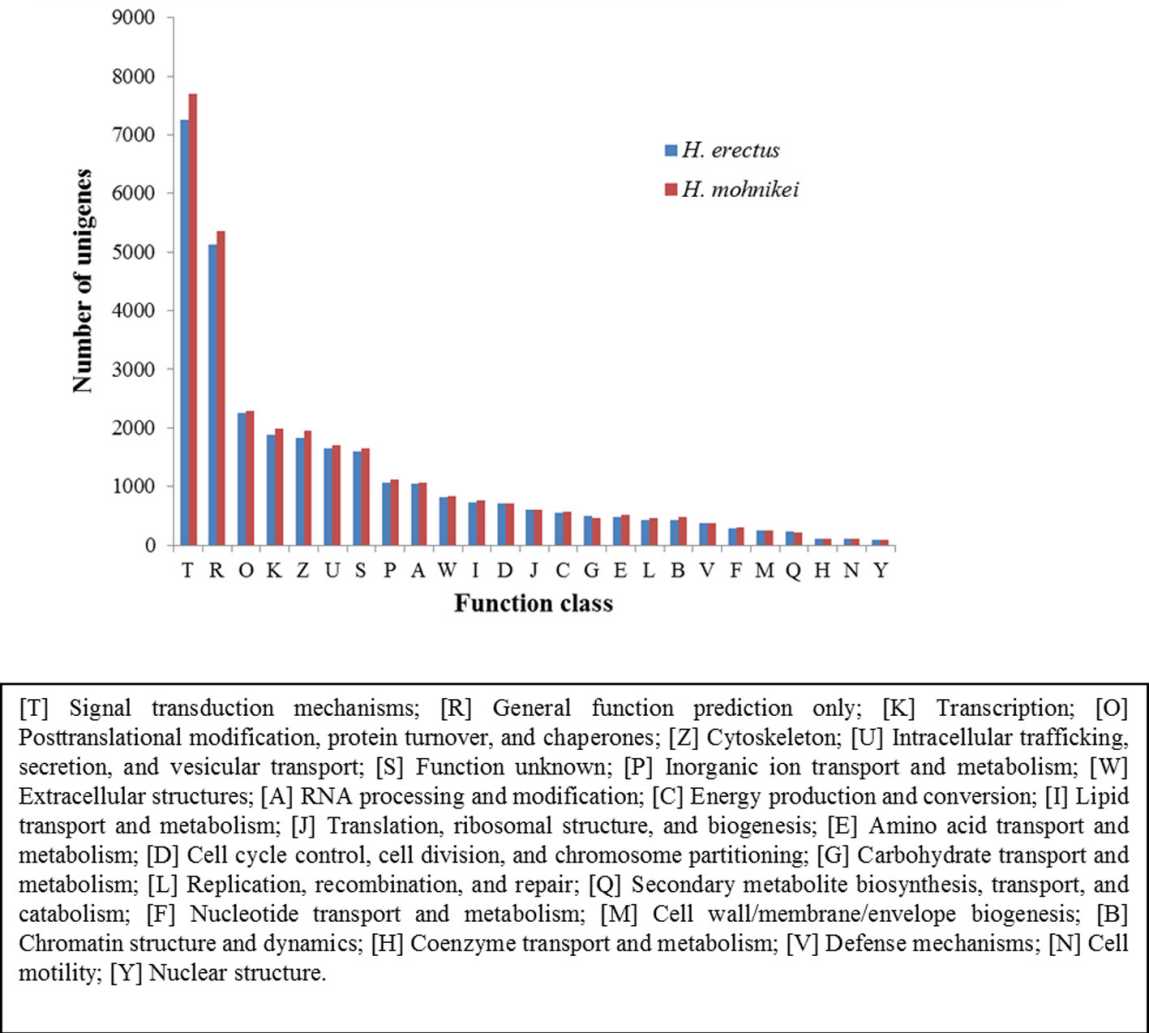
The KOG classifications of unigenes for *H*. *erectus* and *H*. *mohnikei*.

All unigenes were subjected to a search against the KOG database for functional prediction and classification. In total, 13,169 unigenes for *H*. *erectus* and 13,607 unigenes for *H*. *mohnikei* were annotated and both grouped into 25 KOG classifications (Fig 4). However, some of these unigenes were assigned to multiple KOG classifications, and altogether 26,679 and 27,737 functional annotations were obtained for *H*. *erectus* and *H*. *mohnikei*, respectively. Among the 25 KOG categories, the cluster for signal transduction mechanisms [T] was the largest group in both tested species, with 7,264 in *H*. *erectus* and 7,694 in *H*. *mohnikei*, respectively.

### Metabolic pathways by KEGG analysis

**Fig 5:**
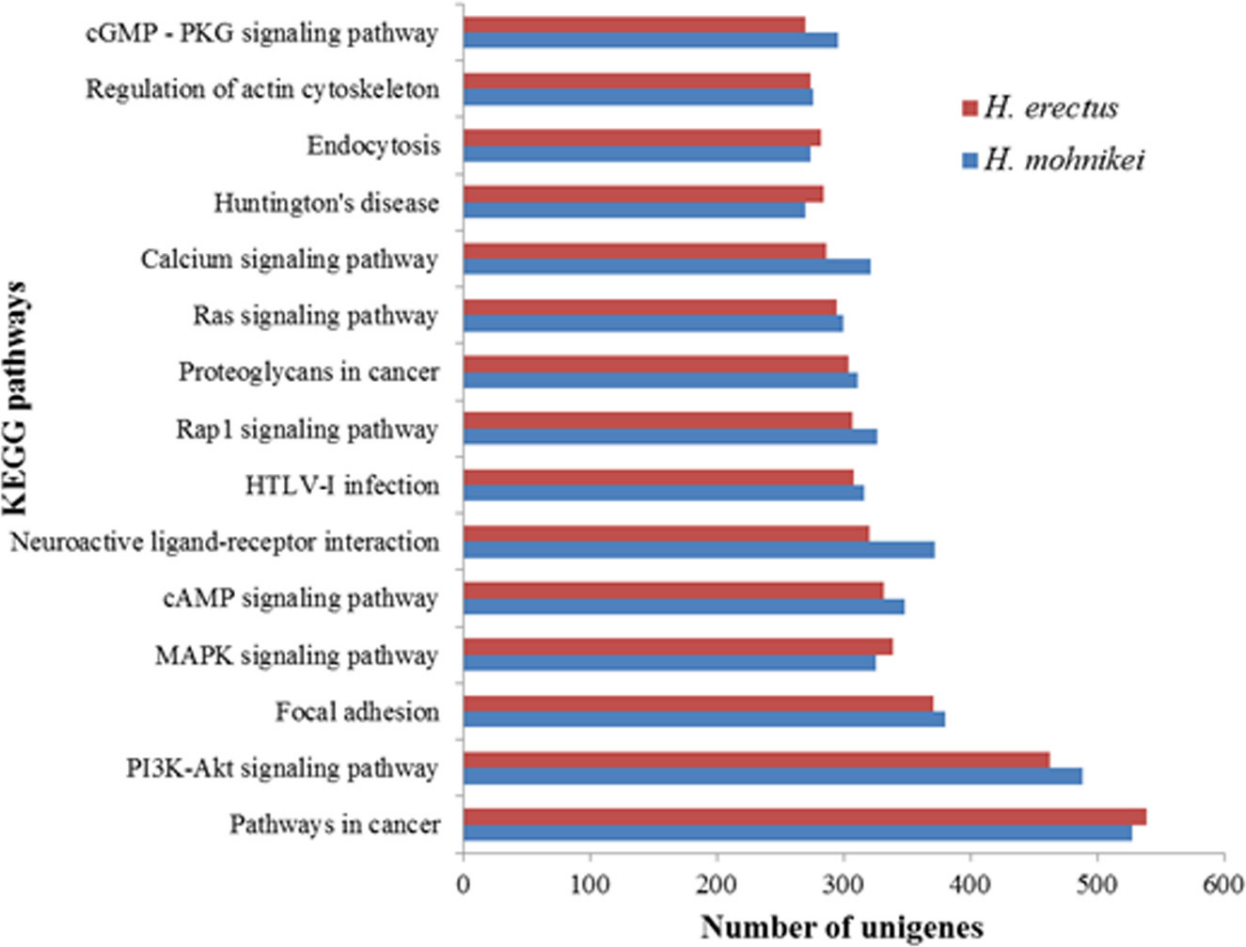
Top 15 KEGG pathways with the highest sequence numbers in *H*. *erectus* and *H*. *mohnikei*.

To identify the biological pathways in seahorses, unigenes were compared against the KEGG database and the corresponding pathways were established. Among the 37,506 unigenes of *H*. *erectus*, 6,403 (17.07%) had significant matches in the database and were assigned to 334 pathways. The pathway in cancer was involved in most unigenes (539), followed by PI3K-Akt signaling pathway (463), focal adhesion (371), MAPK signaling pathway (339), and cAMP signaling pathway (332) (Fig 5; S4 File). Meanwhile, 6,601 (18.27%) unigenes of *H*. *mohnikei* were annotated to 335 pathways. The top three pathways with the greatest number of unigenes were the same to *H*. *erectus*, with pathways in cancer (528), PI3K-Akt signaling pathway (489) and focal adhesion (380) (Fig 5; S4 File).

**Fig 6:**
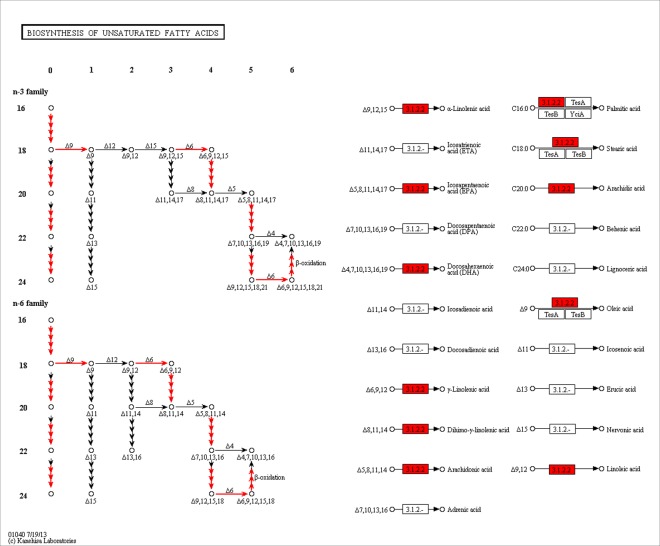
Biosynthesis of unsaturated fatty acids pathways identified in *H*. *erectus*. Red boxes represent unigenes which are annotated to this pathway. Biosynthesis of unsaturated fatty acids pathways identified in *H*. *mohnikei* is the same as *H*. *erectus*.

To further understand the molecular interaction and reaction networks, the pathways of biosynthesis of unsaturated fatty acids in *H*. *erectus* and *H*. *mohnikei* were assigned based on KEGG database. Twenty-four and twenty-one unigenes in *H*. *erectus* and *H*. *mohnikei* were annotated to this pathway, respectively (S5 File). These unigenes encode 14 key enzymes involved in this pathways, including 3-oxoacyl-[acyl-carrier protein] reductase, enoyl-CoA hydratase/long-chain 3-hydroxyacyl-CoA dehydrogenase and 3-oxoacyl-[acyl-carrier protein] reductase. However, seahorses lacked the Δ12 and Δ15 desaturases (Fig 6).

### SSR marker discovery

The potential SSRs were detected in all of the 37,506 assembled unigenes in *H*. *erectus* and 36,113 unigenes in *H*. *mohnikei* using MISA software. Of these unigenes, a total of 8,992 and 9,116 SSRs were identified for *H*. *erectus* and *H*. *mohnikei*, respectively, defined as di-to pentanucleotide SSRs, with a minimum of six repetitions for dinucleotides and five repetitions for the remaining motifs. Of all the motif types, dinucleotides (5,508 for *H*. *erectus* and 5,284 for *H*. *mohnikei*) were the most frequent, followed by tri- (3,142 for *H*. *erectus* and 3,351 for *H*. *mohnikei*) and tetra-nucleotides (328 for *H*. *erectus* and 460 for *H*. *mohnikei*) (Table 4).

**Table 4:** Characteristics of SSRs in *H*. *erectus and H*. *mohnikei*.

		Number of motif repeats					Total
Seahorse species		Di-	Tri-	Tetra-	Penta-	Hexa-	
	Number	5,508	3,142	328	10	4	8,992
*H*. *erectus*	Length of motif	15.86	16.95	20.85	25.00	34.50	
	Proportion	61.25%	34.94%	3.65%	0.11%	0.04%	
	Number	5,284	3,351	460	13	8	9,116
*H*. *mohnikei*	Length of motif	16.02	16.96	20.54	19.23	24.75	
	Proportion	57.96%	36.76%	5.05%	0.14%	0.09%	

**Fig 7:**
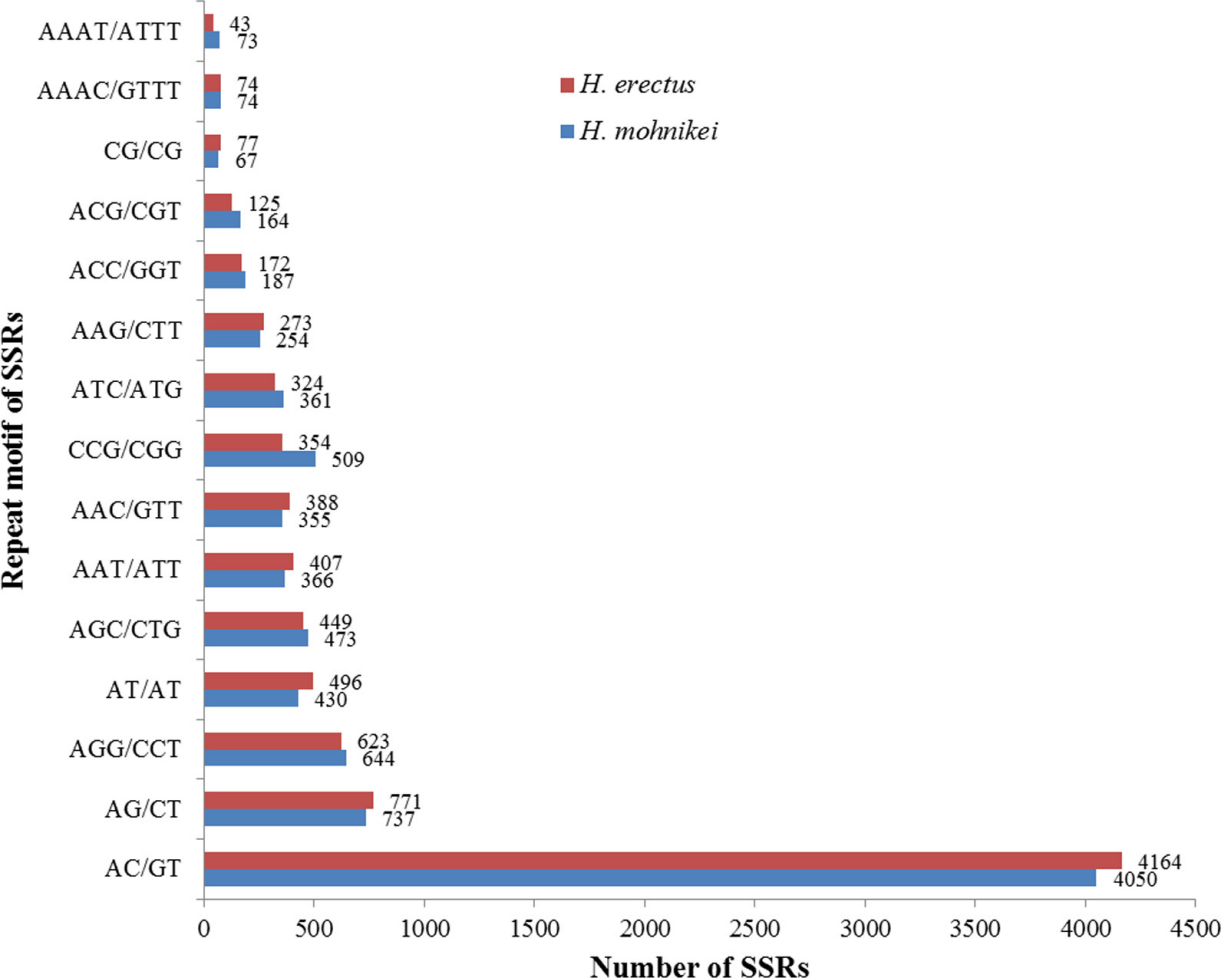
Characteristics of top 15 dominant SSR motifs in *H*. *erectus and H*. *mohnikei*.

Among the microsatellites detected, the dominant classes of repeating sequences in the unigenes of both species were AC/GT (4,050 for *H*. *erectus* and 4,164 for *H*. *mohnikei*), followed by AG/CT (737 for *H*. *erectus* and 771 for *H*. *mohnikei*) and AAG/ CTT (644 for *H*. *erectus* and 623 for *H*. *mohnikei*) repeats (Fig 7).

### SSRs identification and cross-amplification

In order to develop new molecular markers for seahorses, all the unigenes contained microsatellites were used to design primers. A total of 4,023 (44.7%) and 4,511 (49.5%) microsatellites could successfully design the primer pairs for PCR amplification using MISA software (S1 Table). In this study, 129 (including 57 di-, 45 tri-, and 27 tetra-nucleotide repeats) SSR loci of *H*. *erectus* and 110 (48 di-, 40 tri-, and 22 tetra-nucleotide repeats) of *H*. *mohnikei* were randomly selected to check the successful amplification proportion of these SSRs. The results showed that 69 (53.49%) and 56 (50.91%) primer pairs of *H*. *erectus* and *H*. *mohnikei*, respectively, successfully amplified fragments; however, just 20 (15.50%) and 17 (15.45%) showed polymorphism in the tested populations for *H*. *erectus* and *H*. *mohnikei*, respectively, (S6 File; S7 File).

To test the cross utility of markers developed in this study, polymorphic markers from *H*. *erectus* and *H*. *mohnikei* were cross-amplified in each other and also tested in another related species, *H*. *trimaculatus*. Out of the 20 polymorphic SSRs loci from *H*. *erectus*, 14 (70.00%) and 16 (80.00%) were successfully amplified, and 4 (20.00%) and 8 (40.00%) showed polymorphism in *H*. *mohnikei* and *H*. *trimaculatus*, respectively (S1 Table). In addition, out of the 17 polymorphic SSRs loci of *H*. *mohnikei*, 10 (58.82%) and 10 (58.82%) were successfully amplified and 2 (11.76%) and 4 (23.53%) showed polymorphism in *H*. *erectus* and *H*. *trimaculatus*, respectively (S1 Table).

### Genetic diversity in seahorse populations

In the *H*. *erectus* cultured population, the number of alleles (*N*_*A*_) per locus varied from 2 to 8, with a mean value of 4.65. The mean observed (*Ho*) and expected heterozygosity (*He*) were 0.53 and 0.62, respectively. The PIC values were ranged from 0.28 to 0.77, with a mean value of 0.56. In the Yangmadao population of *H*. *mohnikei*, the *Ho* and *He* values were ranged from 0.22 and 0.863, and from 0.17 and 0.84, respectively. The mean *N*_*A*_ was found to be 4.06, and the mean PIC value was 0.48. In the *H*. *trimaculatus* population along China’s coast, the n value of *Ho* and *He* was ranged from 0.48 to 0.79 and from 0.52 to 0.71, respectively, and the mean *N*_*A*_ was 3.25, respectively. It was found that 10 loci out of 20 polymorphic SSRs loci for *H*. *erectus* deviated significantly from the Hardy-Weinberg equilibrium in its population (*P* < 0.05), as well as 5 loci out of 17 polymorphic SSRs loci for *H*. *mohnikei* and 4 loci out of 8 polymorphic SSRs loci for *H*. *trimaculatus* (S1 Table).

## Discussion

The transcriptome exhibits the complete expressed RNA unigenes in the cell, and its characterization would be essential to understand the functional complexity of an organism [16]. The present study reported the transcriptome databases for seahorses *H*. *erectus* and *H*. *mohnikei*. Due to the unavailable of reference genome, the reads produced by Illumina HiSeq ^TM^ 2000 (paired-end reads with 100 bp) were assembled using the *de novo* assembler Trinity [29]. The assembly results indicated that the length distribution pattern and mean length of unigenes (1,317.47 bp for *H*. *erectus* and 1,334.83 bp for *H*. *mohnikei*) were longer than those of recently studied aquatic species, such as Nile tilapia (*Oreochromis niloticus*) (618 bp) [34], Eastern Oyster (*Crassostrea virginica*) (1,023 bp) [35] and blunt snout bream (*Megalobrama amblycephala*) (692.9 bp) [36]. These results suggested that the transcriptome sequencing data for *H*. *erectus* and *H*. *mohnikei* in the present study were well assembled.

The values of annotation rate of unigenes for *H*. *erectus* (46.23%) for *H*. *mohnikei* (49.57%) based on the information available from public databases were similar to the other previously reported fish species, such as rohu carp (*Labeo rohita*) (47.45%) [37], mud loach (*Misgurnus anguillicaudatus*) (43.76%) [38]; while much lower than silver carp (*Hypophthalmichthys molitrix*) (63.2%) [39]. Many studies have suggested that the longer sequences were more likely to obtain BLAST matches in the protein databases [40, 41]. In our study, approximately 80% of unigenes over 1,000 bp in length in the both species had BLAST matches against the Nr database; however, only about 25% of unigenes with lengths shorter than 1,000 bp in the both species generated BLAST matches, which maybe too short to obtain statistically meaningful matches. Meanwhile, for some unigenes, the absence of homologous sequences in the public databases may suggest their possible specific roles in seahorses. Moreover, it was found that the unigenes from both tested species had the best matches with *M*. *zebra* and *O*. *niloticus*, which both belonged to cichlid fish species. This result was consistent with the ray-finned fish phylogenetic tree based on nine nuclear genes [42]. We believe that the large-scale sequencing efforts on seahorse genome in the near future, as well as transcriptome will increase the coverage of our dataset even further.

Gene annotation and pathway analyses are helpful to predict potential genes and their possible functions at a transcriptome level. GO annotation and KOG classification analyses showed that the unigenes from both species presented a similar transcriptome, indicating that these two seahorses were closely genetically-related. Gene function categories associated with cellular processes, cells, and bindings are highly represented in both transcriptomes. These results are similarly found in *O*. *mykiss* [43] and *H*. *molitrix* [39]. Based on the KEGG pathway database, organismal systems represented the dominant pathway and contained 9 different subcategories, mainly including the nervous system, sensory system, immune system, excretory system, endocrine system, and system digestive system. These results indicated a large amount of genes involved in maintaining ordinary physiological function. It is unclear whether or not the pathway in human cancer is involved in a mass of unigenes in both seahorse species, which is similar to clawed frog (*Xenopus tropicalis*) [44] and frog (*Bombina maxima*) [45]. These results would provide the basic information for future studies, such as gene cloning, expression analysis and gene-associated markers identification.

Seahorse has high medicinal effect partly because of high content of highly unsaturated fatty acids (PUFA). Like other marine fish [46, 47], seahorse also lacks the Δ12 and Δ15 desaturases and so cannot form linoleic (18:2n-6) and α-linolenic (18:3n-3) acids from 18:1n-9. The biochemistry of PUFA synthesis, including pathways and reaction mechanisms, has been well described in fish [48]; however, until recently, little is known of the genes and gene products involved and of the factors affecting their expression in seahorse. In this study, many genes, which code the key enzymes in the pathway of biosynthesis of unsaturated fatty acids, were identified by blast against KEEG database, promoting the research on the metabolism and gene regulation of unsaturated fatty acids in seahorses and in other fish species.

Based on the transcriptome data, dinucleotide repeats were the most frequent SSR motif type in both *H*. *erectus* and *H*. *mohnikei*. This finding is consistent with results reported for channel catfish (*Ictalurus punctatus*) [49] and *M*. *amblycephala* [15]. Among the dinucleotide repeats, AC is the most frequent motif in our dataset, which was the same as *I*. *punctatus* [49], Japanese pufferfish (*Fugu rubripes*) [50], okaloosa darter (*Etheostoma okaloosae*) [51], and Tarim schizothoracin (*Schizothorax biddulphi*) [52]. Polymorphic SSR markers have become as one of the most popular genetic markers in some applications, such as in genetic diversity analysis, the genetic maps construction, quantitative trait loci mapping, marker assisted selection breeding and comparative genomics [49, 53]. Development of SSR markers is becoming more and more important with the tremendous development and rapid cost reduction of high through-put sequencing technology, which is because high through-put transcriptome sequencing not only produces an amount of sequence data for marker identification, but also because the obtained markers are gene-based [53]. The markers developed from transcriptome data are advantageous for their functional variation and useful for associated genetic studies [54]. So far, only a few SSR markers have been developed for seahorses [23–25, 55]. In the present study, a large amount of sequences containing SSR loci were obtained, and dozens of SSR markers were subsequently developed within a short time period. Generally, EST-derived SSR are more transferable between species than random genomic SSRs [56], and cross-species amplification is both fast and inexpensive [55]. As the results showed in the present study, a middle rate of cross-amplification was found among the three seahorse species, suggesting the possibility of using primers inter-specifically among seahorse species.

The evaluation of genetic diversity might provide a guide for germplasm resource protection and breeding for seahorses. In the present study, the *Ho* values and *He* of the three seahorses were medium. This result was similar to the three populations of *H*. *trimaculatus* along the coasts of India by using 12 SSRs [25]. In addition, a large proportion of loci were found to deviate significantly from the Hardy-Weinberg equilibrium in this study. Singh also detected significant deviation in allele frequencies from Hardy-Weinberg equilibrium at a few loci [25]. The reasons for deviation from Hardy-Weinberg equilibrium in both researches were deficiency of heterozygotes at these loci. This deficiency of heterozygotes may due to the high inbreeding rate, relatively weak ability of swimming/mobility [1], and special mating pattern, i.e., “monogamy” [57]. Moreover, as seahorses are particularly vulnerable to human activities because of their lengthy durations of parental care, small brood sizes and faithful pair bonds [1], and the interference of human activities in near-coast waters, such as overfishing, habitat destruction and environment pollution, has a serious influence on the seahorse population genetic structure [22].

## Conclusion

This study addressed the application of high through-put sequencing technology for seahorse *de novo* transcriptome sequencing and the most comprehensive study of seahorse transcriptome data. The large number of assembled unigenes and detected SSR markers obtained from the seahorse transcriptome indicated that Illumina paired-end sequencing could be used as a cost-effective and fast approach to discover novel genes and molecular markers for non-model organisms. Based on the sequences with SSR marker, dozens of SSR makers were developed, and they could be utilized in genetic diversity analysis and MAS. In addition, the transcriptome resources available for seahorses would facilitate the conservation management of the seahorse species through a better understanding of its biology, genomics, as well as comparative genome analysis within the *Hippocampus* genus.

## Supporting Information

S1 File The annotation of unigenes of *H*. *erectus* and *H*. *mohnikei*. The unigenes annotated based on the information available from public databases including NCBI nor-redundant protein (Nr), Swiss-Prot protein, eukaryotic Orthologous Groups (KOG), and the Kyoto Encyclopedia of Genes and Genomes (KEGG) for *H*. *erectus* (Table A in S1 File) and *H*. *mohnikei* (Table B in S1 File).
(XLS)

S2 File The candidate orthologous genes for *H*. *erectus* and *H*. *mohnikei* (Table A in S2 File). (XLS)

S3 File GO annotations for *H*. *erectus* and *H*. *mohnikei*. The file contains 14,645 unigenes with a total of 11,484 term occurrences for *H*. *erectus* (Table A in S3 File), and 15,197 unigenes with a total of 11,543 term occurrences for *H*. *mohnikei* (Table B in S3 File) assigned to one or more GO terms.
(XLS)

S4 File Unigenes of *H*. *erectus* and *H*. *mohnikei* mapped to the KEGG pathway database. The file contains 6,403 unigenes for *H*. *erectus* (Table A in S4 File) and 6,601 unigenes for *H*. *mohnikei* (Table B in S4 File) mapped to the KEGG pathway database.
(XLS)

S5 File Unigenes annotated to biosynthesis of unsaturated fatty acids pathways in *H*. *erectus* (Table A in S5 File) and *H*. *mohnikei* (Table B in S5 File). (DOCX)

S6 File The designed primer for the detected SSRs of *H*. *erectus* (Table A in S6 File) and *H*. *mohnikei* (Table B in S6 File). (XLS)

S7 File Part of the page photos of SSR markers identified in this study. (DOCX)

S1 Table Characteristics of SSR isolated from *H*. *erectus* and *H*. *mohnikei*, and application in genetic diversity analysis. Characteristics of 20 polymorphic microsatellite loci isolated from *H*. *erectus and cross-amplification in H*. *mohnikei and H*. *trimaculatus*, *and population genetic diversity of H*. *erectus* (Table A in S1 Table); Characteristics of 17 polymorphic microsatellite loci isolated from *H*. *mohnikei and cross-amplification in H*. *erectus and H*. *trimaculatus*, *and population genetic diversity of H*. *mohnikei* (Table B in S1 Table); The population genetic diversity of *H*. *trimaculatus* (Table C in S1 Table).
(DOCX)
