# Supplementary material for: De Novo Transcriptome Analysis of Two Seahorse Species (Hippocampus erectus and H. mohnikei) and the Development of Molecular Markers for Population Genetics
Source: PLoS One. 2016 Apr 29;11(4):e0154096. doi: 10.1371/journal.pone.0154096 (PMC4851356; doi:10.1371/journal.pone.0154096)
Supplement: S5 File — (DOCX) [file pone.0154096.s005.docx]

**Table A. Unigenes annotated to biosynthesis of unsaturated fatty acids pathways in *H. erectus*.**

Twenty-four unigenes annotated to biosynthesis of unsaturated fatty acids pathways in *H. erectus*. The unigene ID is followed:

comp2079_c0_seq1, comp5146_c0_seq1, comp5241_c0_seq1, comp5891_c0_seq1, comp6027_c0_seq1, comp13505_c0_seq2, comp15062_c0_seq2, comp15063_c0_seq2, comp17158_c0_seq1, comp17676_c0_seq1, comp17760_c0_seq1, comp19540_c0_seq1, comp21810_c0_seq4, comp22230_c1_seq2, comp23324_c0_seq1, comp24683_c2_seq3, comp24751_c0_seq5, comp45496_c0_seq1, comp46108_c0_seq1, comp57578_c0_seq1, comp65197_c0_seq1, comp67307_c0_seq1, comp82778_c0_seq1, comp110846_c0_seq1.

**Table B. Unigenes annotated to biosynthesis of unsaturated fatty acids pathways in *H. mohnikei*.**

Twenty-one unigenes annotated to biosynthesis of unsaturated fatty acids pathways in *H. mohnikei*. The unigene ID is followed:

comp5326_c0_seq1, comp14808_c0_seq1, comp15576_c0_seq1, comp16572_c0_seq1, comp17846_c0_seq1, comp18105_c0_seq4, comp19756_c0_seq4, comp21394_c0_seq1, comp21516_c0_seq2, comp21848_c0_seq2, comp22832_c0_seq1, comp24138_c0_seq3, comp24689_c0_seq1, comp24871_c1_seq1, comp28359_c0_seq1, comp29227_c1_seq5, comp30049_c0_seq1, comp31266_c0_seq1, comp34701_c0_seq1, comp239287_c0_seq1, comp286582_c0_seq1,
