# Supplementary figures and images for: De Novo Transcriptome Analysis of Two Seahorse Species (Hippocampus erectus and H. mohnikei) and the Development of Molecular Markers for Population Genetics
Source: PLoS One. 2016 Apr 29;11(4):e0154096. doi: 10.1371/journal.pone.0154096 (PMC4851356; doi:10.1371/journal.pone.0154096)

S7 File: Part of the page photos of SSR markers identified in this study


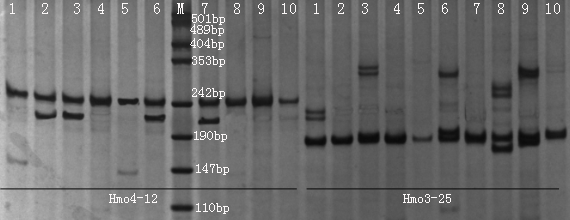


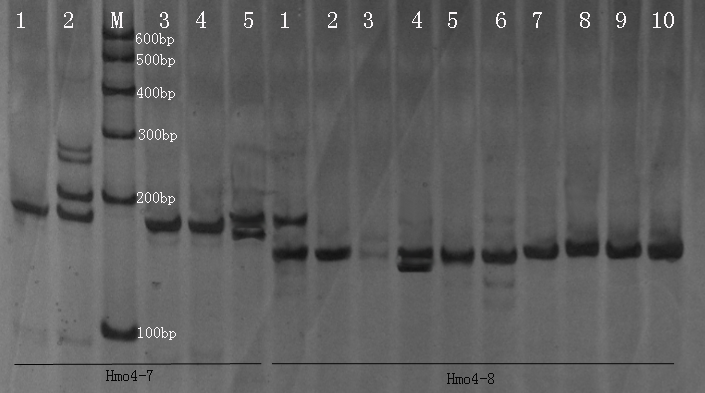


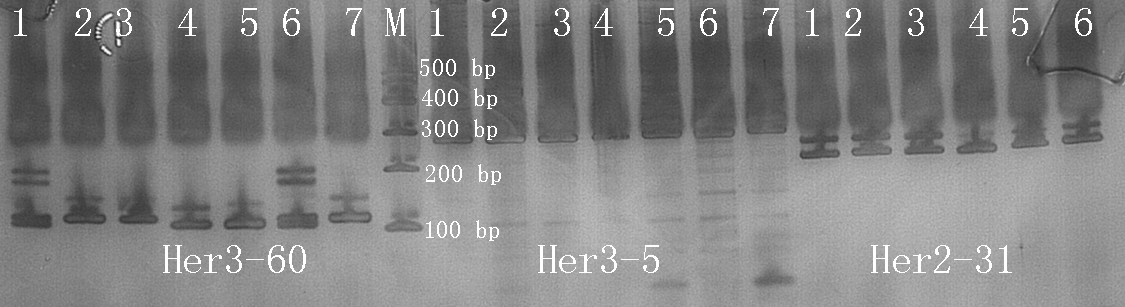







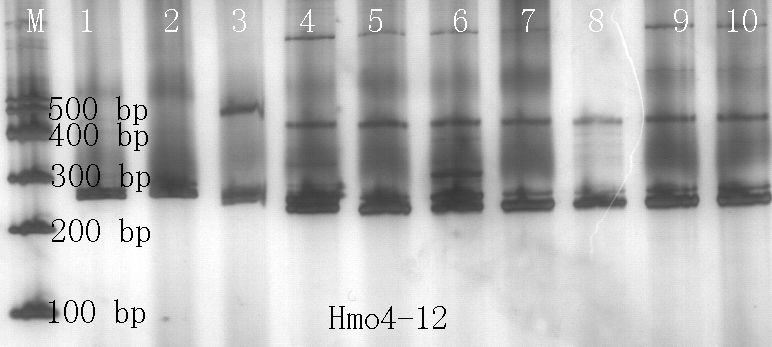

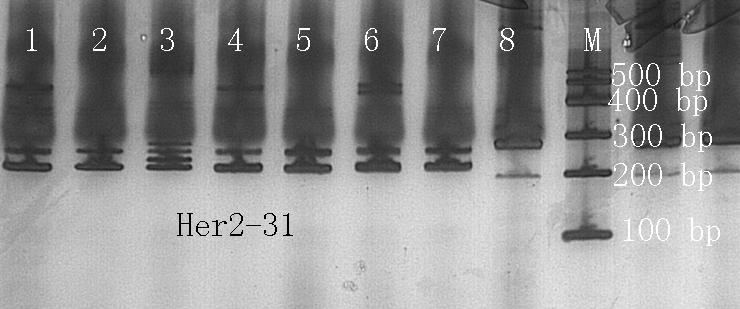

Supplement: S7 File — (DOCX) [file pone.0154096.s007.docx]
