# Supplementary material for: De Novo Transcriptome Analysis of Two Seahorse Species (Hippocampus erectus and H. mohnikei) and the Development of Molecular Markers for Population Genetics
Source: PLoS One. 2016 Apr 29;11(4):e0154096. doi: 10.1371/journal.pone.0154096 (PMC4851356; doi:10.1371/journal.pone.0154096)
Supplement: S1 Table — Characteristics of 20 polymorphic microsatellite loci isolated from H. erectus and cross-amplification in H. mohnikei and H. trimaculatus, and population genetic diversity of H. erectus (Table A in S1 Table); Characteristics of 17 polymorphic microsatellite loci isolated from H. mohnikei and cross-amplification in H. erectus and H. trimaculatus, and population genetic diversity of H. mohnikei (Table B in S1 Table); The population genetic diversity of H. trimaculatus (Table C in S1 Table). (DOCX) [file pone.0154096.s008.docx]

**Table A. Characteristics of 20 polymorphic microsatellite loci** **isolated from *H. erectus and cross-amplification in H. mohnikei and H. trimaculatus*, and population genetic diversity of *H. erectus***

| Locus | Primer sequence (5'→3') | Ta (°C) | Repeat motif | Size range (bp) | *N_A_* | *N_E_* | *H_O_* | *H_E_* | PIC | *P*_HWE_ | *Na* in *H. mo* | *Na* in *H. tr* |
| --- | --- | --- | --- | --- | --- | --- | --- | --- | --- | --- | --- | --- |
| Her2-4 | F: GGGGCTGTTCTTGTCCT | 56 | (GT)_7_ | 162-170 | 5 | 3.95 | 0.42 | 0.65 | 0.6 | 0 | 4 |  |
|  | R: CTGGCTTCGTGTCTTCC |  |  |  |  |  |  |  |  |  |  |  |
| Her3-58 | F: CGGCGACTGCTGCTAC | 48 | (GGC)_5_ | 160-190 | 6 | 4.36 | 0.5 | 0.77 | 0.74 | 0 |  |  |
|  | R: CGAGACGGCGATGAAG |  |  |  |  |  |  |  |  |  |  |  |
| Her2-6 | F: CTCGTCGTTGGGATTGAAG | 50 | (ACC)_8_ | 220-236 | 8 | 5.54 | 0.58 | 0.72 | 0.69 | 0.29 |  | 2 |
|  | R: TTCATCCCCGCCTTTGT |  |  |  |  |  |  |  |  |  |  |  |
| Her3-59 | F: CCGCAGTCGTGACCTCC | 54 | (GGC)_5_ | 240-286 | 5 | 4.43 | 0.67 | 0.67 | 0.74 | 0.36 |  | 2 |
|  | R: CGGGGCTTGCCTTGTG |  |  |  |  |  |  |  |  |  |  |  |
| Her3-60 | F: GTTCAAAGGTCCAGCAGA | 50 | (TCT)_7_ | 116-130 | 3 | 2.27 | 0.67 | 0.56 | 0.47 | 0.91 |  |  |
|  | R: AGATGAGGACGACGAGGA |  |  |  |  |  |  |  |  |  |  |  |
| Her2-19 | F: GAGAAAAGCGAGCAAAT | 50 | (TA)_10_ | 100-116 | 2 | 1.88 | 0.42 | 0.47 | 0.36 | 0.59 |  | 2 |
|  | R: AAGGAAAACAAAATGGC |  |  |  |  |  |  |  |  |  |  |  |
| Her2-21 | F:GATGACCTTGAAATAGCTG | 50 | (ATT)_6_ | 140-146 | 3 | 2.46 | 0.25 | 0.59 | 0.42 | 0 | 2 | 2 |
|  | R:TATTGGACCCGTGACTC |  |  |  |  |  |  |  |  |  |  |  |
| Her2-24 | F: AACAACGCAAGAAAGATG | 50 | (AC)_9_ | 152-166 | 2 | 1.95 | 0.73 | 0.49 | 0.37 | 0.02 |  | 2 |
|  | R:TTCAAGGCAATACTCAATAA |  |  |  |  |  |  |  |  |  |  |  |
| Her2-25 | F: CAGTCATTGTTTGCTTCTT | 48 | (GT)_9_ | 174-180 | 4 | 2.67 | 0.58 | 0.63 | 0.36 | 0.33 | 2 |  |
|  | R: CCATCAGTCTCCTTTTCA |  |  |  |  |  |  |  |  |  |  |  |
| Her2-31 | F: TGCTCCGTTCTCCTTTG | 48 | (AT)_9_ | 218-240 | 4 | 1.42 | 0.33 | 0.3 | 0.28 | 1 |  | 4 |
|  | R: CTGGATAAATGCCTTGGT |  |  |  |  |  |  |  |  |  |  |  |
| Her2-35 | F: TCTTGTGGGAGCGGTTTC | 48 | (AC)_9_ | 168-200 | 6 | 4.24 | 0.75 | 0.76 | 0.73 | 0 | 5 | 5 |
|  | R: CCATGTGATTTTGTCCTTGTT |  |  |  |  |  |  |  |  |  |  |  |
| Her2-44 | F: TAGTGGTGGCAACTTCAG | 50 | (AC)_9_ | 240-280 | 7 | 5.05 | 0.75 | 0.7 | 0.57 | 0.81 |  |  |
|  | R: CACAGCAGACTCTTCCAG |  |  |  |  |  |  |  |  |  |  |  |
| Her2-46 | F: GCGGAGCCTGAACTA | 50 | (CA)_7_ | 180-220 | 4 | 2.38 | 0.58 | 0.58 | 0.42 | 0.57 |  |  |
|  | R: ATCTACGCAACAAATGG |  |  |  |  |  |  |  |  |  |  |  |
| Her2-55 | F: CAAATCACATGACGGAGAC | 50 | (TG)_5_ | 240-256 | 4 | 3.03 | 0.75 | 0.67 | 0.51 | 0.1 |  |  |
|  | R: CGTACTATGCGTATGACTAAAT |  |  |  |  |  |  |  |  |  |  |  |
| Her3-5 | F: GAAGCAGAGGAGTGCGTGTC | 58 | (GGT)_8_ | 280-298 | 6 | 4.36 | 0.5 | 0.77 | 0.74 | 0 |  |  |
|  | R: TTCTCGTCGTTGGGATTGA |  |  |  |  |  |  |  |  |  |  |  |
| Her3-19 | F: AGCAAACGGAAGCACAAG | 56 | (AGC)_7_ | 107-40 | 7 | 4.8 | 0.5 | 0.69 | 0.77 | 0 |  |  |
|  | R: GAAGTTGCCGTCGGAGTC |  |  |  |  |  |  |  |  |  |  |  |
| Her4-5 | F:GGGAAGTGATGACAGCGTGAA | 52 | (ATCC)_6_ | 116-132 | 5 | 3.74 | 0.67 | 0.73 | 0.68 | 0.06 |  | 4 |
|  | R: AGGGTCGGAGCAGGTTGA |  |  |  |  |  |  |  |  |  |  |  |
| Her4-10 | F: TGGAACCAGGCGTCTACC | 48 | (GACA)_5_ | 113-121 | 3 | 2.13 | 0.25 | 0.53 | 0.47 | 0.01 |  |  |
|  | R: AGCCACCCACTGACCTTT |  |  |  |  |  |  |  |  |  |  |  |
| Her4-18 | F: CACGCAACCTGCATCCCT | 48 | (CAAG)_5_ | 153-160 | 4 | 2.64 | 0.25 | 0.62 | 0.57 | 0 |  |  |
|  | R: CAGCCCCGTCGTCAAACT |  |  |  |  |  |  |  |  |  |  |  |
| Her4-23 | F: AGGCTGAAAATGAGAACC | 48 | (ATTG)_5_ | 217-229 | 4 | 3.06 | 0.33 | 0.57 | 0.62 | 0.01 |  |  |
|  | R: CGCAGAAATGAAGATGGA |  |  |  |  |  |  |  |  |  |  |  |
| Mean value |  |  |  |  | 4.65 | 3.35 | 0.62 | 0.66 | 0.56 |  |  |  |

**Table B. Characteristics of 17 polymorphic microsatellite loci isolated from *H. mohnikei and cross-amplification in H. erectus and H. trimaculatus*, and population genetic diversity of *H. mohnikei***

| Locus | Primer sequence (5'→3') | Ta (°C) | Repeat motif | Size range (bp) | *N_A_* | *N_E_* | *H_O_* | *H_E_* | PIC | *P*_HWE_ | *Na* in *H. er* | *Na* in *H. tr* |
| --- | --- | --- | --- | --- | --- | --- | --- | --- | --- | --- | --- | --- |
| Hmo2-23 | F: CTGCCCAATGGTATTATTAAA | 58 | (GCCA)_3_ | 170-190 | 2 | 1.48 | 0.41 | 0.32 | 0.27 | 0.21 |  |  |
|  | R: ACGGTCTTAATTTATGCAGGT |  |  |  |  |  |  |  |  |  |  |  |
| Hmo2-26 | F: GCAAACCTGTTCAGGATAAG | 58 | (GGAT)_5_ | 136-170 | 2 | 1.84 | 0.6 | 0.46 | 0.35 | 0.01 |  | 2 |
|  | R: CAGAGAGGAGCACAGTTCTAA |  |  |  |  |  |  |  |  |  |  |  |
| Hmo2-30 | F: GTTCATCTTTTTGCAAGTGTC | 56 | (ATTAGC)_3_ | 170-180 | 3 | 2.15 | 0.76 | 0.54 | 0.35 | 0.43 |  | 3 |
|  | R: CTGGGTCATAATCCAGATACA |  |  |  |  |  |  |  |  |  |  |  |
| Hmo2-40 | F: GATGGTGGACAATGAACTCTA | 56 | (GAT)_7_ | 188-210 | 4 | 2.3 | 0.73 | 0.57 | 0.32 | 0.47 | 3 | 3 |
|  | R: AGTTCCCCTCTCTGAGAAGTA |  |  |  |  |  |  |  |  |  |  |  |
| Hmo3-15 | F: CAGCAGTTACTCCTCCAAAG | 48 | (GCT)_7_ | 131-170 | 7 | 2.92 | 0.44 | 0.66 | 0.62 | 0.24 |  |  |
|  | R: GATTCCACCTCTGACCTACC |  |  |  |  |  |  |  |  |  |  |  |
| Hmo3-25 | F: AAAACACGGGCGAGATGA | 58 | (GAT)_7_ | 170-212 | 5 | 1.61 | 0.41 | 0.38 | 0.36 | 0.12 |  |  |
|  | R: GCGAGACTGAGGCACAGAAG |  |  |  |  |  |  |  |  |  |  |  |
| Hmo2-53 | F: ATGTCTGGCTTGGGTCT | 50 | (TC)_8_ | 245-290 | 4 | 2.3 | 0.73 | 0.57 | 0.32 | 0.47 |  |  |
|  | R: CGAGACGGCGATGAAG |  |  |  |  |  |  |  |  |  |  |  |
| Hmo2-54 | F: TAACGGCGTGCCAACT | 52 | (TG)_6_ | 190-210 | 2 | 2 | 0.76 | 0.5 | 0 | 2 |  |  |
|  | R: CACCGCAGCCATAGAA |  |  |  |  |  |  |  |  |  |  |  |
| Hmo4-1 | F: TCACAGAGGCGCTTCTTA | 48 | (TCCG)_10_ | 260-300 | 6 | 2.59 | 0.59 | 0.61 | 0.56 | 0.1 | 3 | 3 |
|  | R: GCAGGAGCAAAACTATTCG |  |  |  |  |  |  |  |  |  |  |  |
| Hmo4-5 | F: CGCCAAAGTCTTCAGCTC | 48 | (CGCT)_6_ | 172-212 | 10 | 6.1 | 0.41 | 0.84 | 0.72 | 0 |  |  |
|  | R: CGGAAAACATCGTCACCC |  |  |  |  |  |  |  |  |  |  |  |
| Hmo4-7 | F: CATGAGCGGACAAACAAA | 58 | (TCGC)_6_ | 186-198 | 2 | 1.2 | 0.19 | 0.17 | 0.64 | 0.15 |  |  |
|  | R: CTGAGACTACCCGAACGAG |  |  |  |  |  |  |  |  |  |  |  |
| Hmo4-8 | F: CCAAACCTGCTACACGACAT | 58 | (ATCG)_6_ | 148-180 | 5 | 1.82 | 0.48 | 0.45 | 0.42 | 0 |  |  |
|  | R: CACAAAGCAAGCAAGAAAGC |  |  |  |  |  |  |  |  |  |  |  |
| Hmo4-12 | F: CCCAAGTGACGCAAAATC | 48 | (GGAT)_5_ | 230-254 | 9 | 5.83 | 0.56 | 0.83 | 0.71 | 0 |  |  |
|  | R: AGTACCAGCGGAAGAAGAAT |  |  |  |  |  |  |  |  |  |  |  |
| Hmo2-52 | F: ATGGAGAAGCATTTGAGTG | 50 | (GT)_6_ | 210-230 | 2 | 2 | 0.76 | 0.5 | 0.37 | 0.08 |  |  |
|  | R: GAAGCAGTTGGCGTTTA |  |  |  |  |  |  |  |  |  |  |  |
| Hmo2-50 | F: TTTGGGGACTCTACCTTT | 50 | (CA)_5_ | 170-190 | 2 | 0.76 | 0.54 | 0.43 | 2.15 | 0 |  |  |
|  | R: TGTCACCGTGGACCTG |  |  |  |  |  |  |  |  |  |  |  |
| Hmo2-55 | F: GCTGCCGAGACTAACC | 54 | (TG)_6_ | 240-260 | 2 | 1.98 | 0.69 | 0.49 | 0 | 2 |  |  |
|  | R: AGAAGGGAGCATTTGG |  |  |  |  |  |  |  |  |  |  |  |
| Hmo2-51 | F: TGGAGAAGCATTTGAGTG | 54 | (GT)_5_ | 210-230 | 2 | 2 | 0.76 | 0.5 | 0.08 | 2 |  |  |
|  | R: GAAGCAGTTGGCGTTTA |  |  |  |  |  |  |  |  |  |  |  |
| Mean value |  |  |  |  | 4.06 | 2.40 | 0.58 | 0.52 | 0.48 |  |  |  |

**Table C. The population genetic diversity of *H. trimaculatus***

| Locus | Size range (bp) | *N_A_* | *N_E_* | *H_O_* | *H_E_* | PIC | *P*_HWE_ |
| --- | --- | --- | --- | --- | --- | --- | --- |
| Her2-19 | 80-116 | 2 | 1.65 | 0.40 | 0.33 | 0.27 | 0.13 |
| Her2-31 | 260-280 | 4 | 2.86 | 0.36 | 0.24 | 0.57 | 0.00 |
| Her2-35 | 140-180 | 5 | 3.58 | 0.74 | 0.76 | 0.63 | 0.49 |
| Her4-5 | 118-134 | 4 | 3.68 | 0.64 | 0.53 | 0.63 | 0.14 |
| Hmo2-26 | 136-160 | 2 | 1.45 | 0.50 | 0.46 | 0.62 | 0.00 |
| Hmo2-30 | 170-192 | 3 | 2.36 | 0.56 | 0.54 | 0.25 | 0.02 |
| Hmo2-40 | 188-240 | 3 | 2.18 | 0.52 | 0.54 | 0.36 | 0.21 |
| Hmo4-1 | 260-300 | 3 | 2.43 | 0.54 | 0.48 | 0.48 | 0.00 |
| Mean value |  | 3.25 | 2.52 | 0.53 | 0.49 | 0.48 |  |
